# Mother and Infant

**Published:** 1884-03

**Authors:** 


### MOTHER AND INFANT.

Of every two children born into the world, one dies before ten years have passed away.

Of every three children born, one dies before five years.

Of every five children born, one dies within a year.

With intelligent care, instead of half of all the children who come into the world dying within ten years, four-fifths of them ought to live, and would live.

That so many die is owing to the fact, in part, that mothers do not know soon enough that anything is the matter with their children, and the time is past for them to be saved; but they can know, they ought to know; and it is proposed here to show the mother how to know promptly that her child is not well, and to determine at once what part of the body suffers or is threatened.

If an infant is well its tongue is white, its eyes are bright, its flesh plump and full, and its skin soft and cool ; the breathing is regular and easy and natural ; when awake it is lively, cheerful, always disposed to laugh, always pleased to be played with ; and, when asleep, it rests quietly, the countenance is composed, and conveys an expression of happy enjoyment.

